# Efficient Light-Driven Hydrogen Evolution Using a Thiosemicarbazone-Nickel (II) Complex

**DOI:** 10.3389/fchem.2019.00405

**Published:** 2019-06-27

**Authors:** Stylianos Panagiotakis, Georgios Landrou, Vasilis Nikolaou, Anisa Putri, Renaud Hardré, Julien Massin, Georgios Charalambidis, Athanassios G. Coutsolelos, Maylis Orio

**Affiliations:** ^1^Laboratory of Bioinorganic Chemistry, Department of Chemistry, University of Crete, Heraklion, Greece; ^2^Aix Marseille Université, CNRS, Centrale Marseille, iSm2, Marseille, France

**Keywords:** light-driven hydrogen production, catalyst, nickel, molecular photosensitizer, photophysics

## Abstract

In the following work, we carried out a systematic study investigating the behavior of a thiosemicarbazone-nickel (II) complex (**NiTSC-OMe**) as a molecular catalyst for photo-induced hydrogen production. A comprehensive comparison regarding the combination of three different chromophores with this catalyst has been performed, using [**Ir(ppy)**_**2**_**(bpy)]PF**_**6**_, **[Ru(bpy)**_**3**_**]Cl**_**2**_ and [**ZnTMePy]PCl**_**4**_ as photosensitizers. Thorough evaluation of the parameters affecting the hydrogen evolution experiments (i.e., concentration, pH, solvent nature, and ratio), has been performed in order to probe the most efficient photocatalytic system, which was comprised by **NiTSC-OMe** and [**Ir(ppy)**_**2**_**(bpy)]PF**_**6**_ as catalyst and chromophore, respectively. The electrochemical together with the photophysical investigation clarified the properties of this photocatalytic system and allowed us to propose a possible reaction mechanism for hydrogen production.

## Introduction

One of the most important challenges of our society, that still lie ahead, is to discover renewable and abundant energy sources (Hosenuzzaman et al., 2015; Hosseini and Wahid, 2016). Solar energy is indeed an attractive and unlimited energy source which nonetheless requires the development of novel as well as efficient storage technologies (Styring, 2012; Tachibana et al., 2012; Faunce et al., 2013). Interestingly, hydrogen could unquestionably be applied for such a purpose: (i) it is the simplest and the most plentiful element on earth, (ii) the energy of the hydrogen-hydrogen bond is high, and (iii) it is considered as a non-polluting fuel (Peel, 2003). Hence, photocatalytic water splitting leading to hydrogen production is a method that without any doubt could be proved as an auspicious solution (Lewis and Nocera, 2006). Photocatalytic hydrogen production can be accomplished by systems containing a photosensitizer, a sacrificial electron donor and a catalyst (Ladomenou et al., 2015; Yuan et al., 2017). Nevertheless, there are plenty unsolved issues that still rest in the field of photocatalytic hydrogen production. Specifically, the development of systems utilizing earth-abundant materials with enhanced efficiency and durability (Wang and Sun, 2010; Du and Eisenberg, 2012). To that end, numerous hydrogen evolution catalysts along with a great number of different photosensitizers have been extensively examined over the last years (Tran et al., 2010, 2012; Du and Eisenberg, 2012; Wang et al., 2012; Sartorel et al., 2013).

Photocatalytic systems involving low-cost molecular catalysts and compounds prepared through easy synthetic approaches have been widely studied over the past decade (Artero et al., 2011; Eckenhoff et al., 2013; Ladomenou et al., 2015). Specifically, cobaloximes (Fihri et al., 2008; Lazarides et al., 2009, 2014; Du and Eisenberg, 2012; Landrou et al., 2016; Panagiotopoulos et al., 2016), and other polypyridine cobalt complexes have been applied as noble-metal-free catalysts (Eckenhoff et al., 2013; Yin et al., 2015; Zee et al., 2015). Although, several of these catalysts are efficient for photocatalytic hydrogen evolution reaction (HER), their stability was greatly limited upon visible light irradiation. Moreover, many researches draw inspiration from Nature trying to replicate the function of the hydrogenase enzymes (Lubitz et al., 2014; Brazzolotto et al., 2016), leading to the design of nickel complexes that were evaluated as molecular catalysts for HER. As a result, plenty nickel catalysts such as nickel bis(diphosphine) (DuBois and DuBois, 2009a,b; Helm et al., 2011; McLaughlin et al., 2011), and pyridinethiolate (Han et al., 2012, 2013; Rao et al., 2016) have been applied in such schemes, since they reproduce the structure of the active site of hydrogenase. Due to the effect of non-innocent ligands, (Han et al., 2012, 2013; Rao et al., 2015, 2016; Inoue et al., 2017) such nickel complexes have displayed excellent efficiency as catalyst reaching around 7,500 TON (Han et al., 2013; Rao et al., 2016). Thiosemicarbazone metal complexes are an emerging class of new HER electrocatalysts (Haddad et al., 2016, 2017; Straistari et al., 2017, 2018a,b) that have already been proved to be redox active (Blanchard et al., 2005; Haddad et al., 2017; Straistari et al., 2017) The presence of S-donors as well as N-atoms in thiosemicarbazone allows the protonation of the ligand and serve as proton relays (Campbell, 1975; DuBois, 2014; Coutard et al., 2016). One of the most essential aspect of light-driven proton reduction is the appropriate choice of the light-harvesting unit (i.e., photosensitizer, Ps). Despite the fact that [Ru(bpy)_3_]Cl_2_ remains the most widely employed chromophore in such systems (Khnayzer et al., 2014; Lo et al., 2016), iridium complexes are still the most efficient entities found in several photocatalytic systems (Goldsmith et al., 2005; Andreiadis et al., 2011). Additionally, porphyrins and other tetrapyrrolic derivatives can be effective candidates for HER due to their unique stability, electrochemical properties, and appropriate energy levels (Ladomenou et al., 2015). For this reason, various metalloporphyrins such as Zn(II) or Sn(IV), have been utilized as photosensitizers for photocatalytic HER over the years (Lazarides et al., 2014; Koposova et al., 2016; Landrou et al., 2016; Queyriaux et al., 2018).

**Figure 1:**
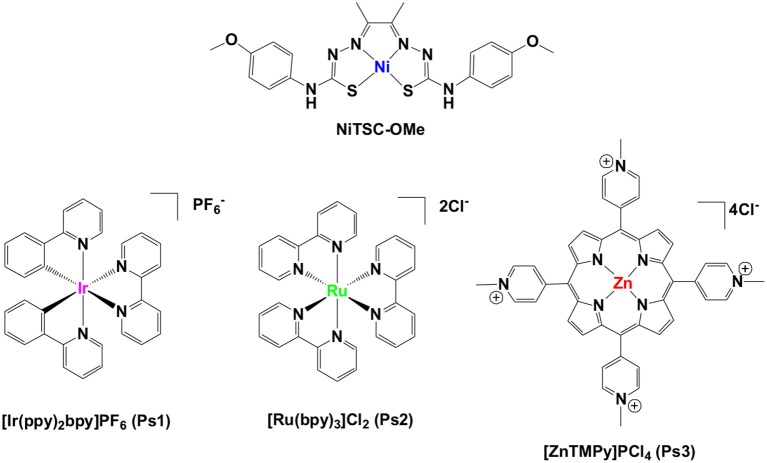
Structures of the molecular catalyst and the photosensitizers used in this work.

Here we will discuss the implications of our findings regarding a novel photoinduced HER scheme using a noble-metal-free *bis*-thiosemicarbazone nickel (II) complex (Straistari et al., 2017), namely **NiTSC-OMe**. In this study, three different light harvesting complexes, **[Ir(ppy)**_**2**_**(bpy)]PF**_**6**_ **(Ps1)**, **[Ru(bpy)**_**3**_**]Cl**_**2**_ (**Ps2**), and [**ZnTMePy]PCl**_**4**_ **(Ps3)** (Andreiadis et al., 2011; Lazarides et al., 2014; Natali et al., 2014) were used as photosensitizers and trimethylamine (TEA) as the sacrificial electron donor (Figure 1). The efficiency of the resulting photocatalytic system was optimized by studying different concentrations of the catalyst, the effect of solvent mixture, the solvent ratio, and the influence of pH in the buffer solution. The electron transfer processes that occur were examined through fluorescence spectroscopic techniques. To solidify the photochemical stability of our system, regeneration experiments were conducted and the homogeneous nature of our catalytic system was proved using poisoning experiments. Based on the results gathered from these studies we were finally able to propose a possible reaction mechanism for light-driven hydrogen production with our photocatalytic system.

## Experimental Section

### Materials and Methods

Reagents and solvents were purchased as reagent grade from usual commercial sources and were used without further purification, unless otherwise stated. **[Ir(ppy)**_**2**_**(bpy)]PF**_**6**_ and [**Ru(bpy)**_**3**_**]Cl**_**2**_ were purchased from commercial sources and used without further purification. The nickel thiosemicarbazone complex (**NiTSC-OMe**) (Straistari et al., 2017) and the Zinc (II) *meso*-tetrakis (1-methylpyridinium-4-yl) porphyrin tetrachloride ([**ZnTMePy]PCl**_**4**_) (Lazarides et al., 2014) were prepared as previously reported.

### Photophysical Measurements

UV-Vis absorption spectra were measured on a Shimadzu UV-1700 spectrophotometer using 10 mm path-length cuvettes (Figure S1). The emission spectra were measured by exciting the samples at 337 nm using a JASCO FP-6500 fluorescence spectrophotometer equipped with a red sensitive WRE-343 photomultiplier tube (wavelength range 200–850 nm).

### Photocatalytic HER Experiments

For the photoinduced HER studies, each sample was prepared in a 42 mL glass vial with silicone septum. The buffer solutions were prepared by dissolving the sacrificial electron donor [triethylamine (TEA) or ascorbic acid (AA)] in water. It was necessary to add a small amount of acetonitrile in order to obtain a homogeneous solution. The pH was adjusted to the required value using concentrated HCl or NaOH solutions. Then the organic solvent (CH_3_CN or EtOH) was added in order to obtain the desired ratio. For the sample preparation, the chromophore was dissolved in the buffer solution and consequently a solution of the catalyst in CH_3_CN or EtOH was added. The final volume of the sample was 5 mL and the mixture was degassed for 10 min using nitrogen. The vials were sealed and the samples irradiated with a white LED lamp (power of 40 W, color temperature of 6,400 K and lumen of 3,800 LM, Figure S2). The amounts of produced hydrogen were determined by gas chromatography (external standard technique) using a Shimadzu GC-2010 plus chromatograph with a TCD detector and a molecular sieve 5 Å column (30–0.53 mm). Gas samples of 100 μL were taken from the headspace and injected immediately into the GC. In all cases, the reported results are the average of three independent experiments. The TONs were calculated using the produced moles of hydrogen vs. the moles of the catalyst. Control experiments were performed under the same conditions after the removal of the catalyst from the hydrogen generating system.

Mercury poisoning experiments were performed, in order to examine the possible formation of metallic nanoparticles or colloids during the hydrogen evolution process. In these studies, an excess of mercury (ca. 40 equiv.) was added to the hydrogen evolution solutions (prepared with the above mentioned procedure).

## Results and Discussion

**Figure 2:**
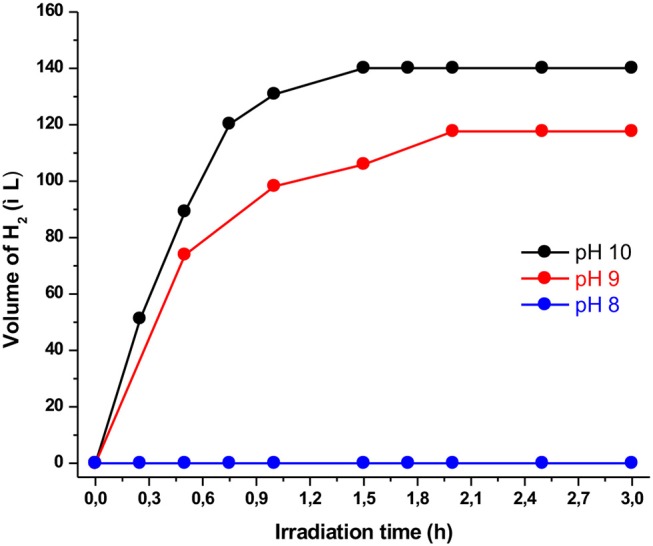
Plot of hydrogen production upon irradiation of solutions (4:1 CH_3_CN/H_2_O) containing **Ps1** (5 × 10^−4^ M), **NiTSC-OMe** (10^−5^ M), and TEA [5% (v/v)] at different pH values.

In our recent work, we reported the synthesis of a novel nickel catalyst (**NiTSC-OMe**, Figure 1) that exhibits high electrocatalytic activity for proton reduction to dihydrogen (Straistari et al., 2017). Based on these encouraging results we wanted to examine the capability of this catalyst toward photochemical hydrogen production. Thus, in the present study three different chromophores (Figure 2) were tested as photosensitizers and combined with the **NiTSC-OMe** catalyst to determine its ability as an effective photocatalytic system to reduce protons into hydrogen. We have analyzed various parameters, such as the concentration of the catalyst (10^−5^ – 5 × 10^−8^ M), the pH of the buffer solution, the effect of the solvent ratio in the photocatalytic mixture and the stability of our system. We carried out several experiments using TEA [5% (v/v)] or AA (0.2 M) as the sacrificial electron donors in various pH buffers (from pH = 2.5 to pH = 10). In addition, different concentrations of the catalyst and the chromophore were tested using varied solvent mixtures. Notably, in all cases no hydrogen production was observed using [**Ru(bpy)**_**3**_**]**^**2+**^ (**Ps2**) or [**ZnTMePy]**^**+**^ (**Ps3**) as photosensitizers. However, when [**Ir(ppy)**_**2**_**(bpy)]**^**+**^ (**Ps1**) was used as photosensitizer and TEA [5% (v/v)] as sacrificial electron donor, a photocatalytic hydrogen evolution of 140 μL from 1 ml of H_2_O was recorded, highlighting once more that the photosensitizer is an essential component in such photocatalytic HER systems.

In the above mentioned system, the first parameter that we examined was the effect of the protons concentration (pH). In detail, we used three different buffer solutions with pH values of 8, 9, and 10, concluding that the optimum one was at pH = 10. As presented in Figure 2 using the solution with pH = 8, no hydrogen production was observed after 3 h of irradiation. On the contrary, using buffer solutions of pH = 9 and pH = 10 we detected hydrogen production of 105 and 125 TONs, respectively. These findings are consistent with results derived from similar systems in which the optimum pH value is close to the pKa value of the sacrificial electron donor. Namely, in our case since the pKa of TEA is 10.7, the optimum pH of the buffer solution was expected at pH = 10 (Pellegrin and Odobel, 2017). Moreover, at pH values lower than its pKa value TEA is protonated and loses its electron donating ability (Rao et al., 2016).

**Figure 3:**
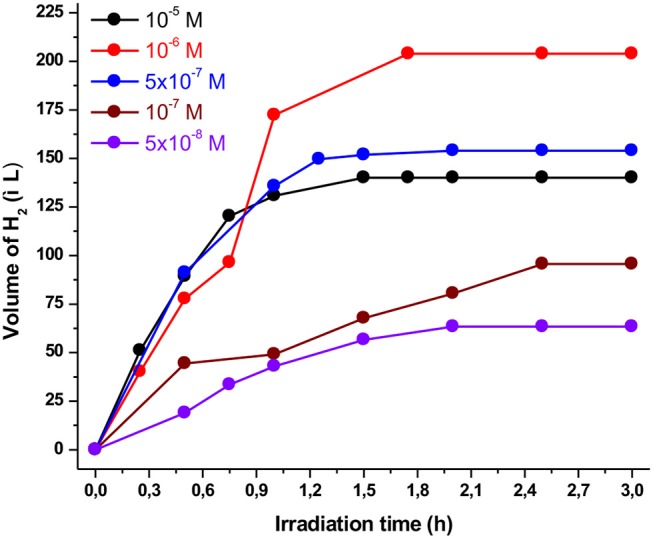
Plot of hydrogen production upon irradiation of different **NiTSC-OMe** concentrations containing **Ps1** (5 × 10^−4^ M), TEA [5% (v/v)] in a 4:1 CH_3_CN/H_2_O solution at pH 10.

Furthermore, the performances of the photocatalytic HER systems strongly depend on the catalyst concentration as well as the relative ratio between the photosensitizer and the catalyst. Accordingly, we kept the concentration of the **Ps1** photosensitizer constant (5 × 10^−4^ M), while the concentration of the catalyst varied from 10^−5^ to 5 × 10^−8^ M. As illustrated in Figure 3, the volume of the produced H_2_ was increased when the concentration of the catalyst decreased from 10^−5^ to 10^−6^ M, reaching a maximum of 204 μL. In addition, further decrease in the concentration of the catalyst (5 × 10^−7^–5 × 10^−8^) resulted in lower catalytic efficiency. On the other hand, the catalytic activity of the system (TON) was increased when the concentration of the catalyst decreased from 10^−5^ to 5 × 10^−8^ M (Figure S4). Notably, when the concentration of the **NiTSC-OMe** was 5 × 10^−8^ M the system displayed the maximum TON and TOF values, namely 11,333 and 7,971, respectively (see Table S1). These results are in contrast with our previous work (Lazarides et al., 2014; Panagiotopoulos et al., 2016), where the maximum photocatalytic activity was observed when the concentration of the catalyst was in excess compared to that of the photosensitizer. This behavior can be attributed to two possible reasons: (i) the quenching of the excited state of the **Ps1** by the **NiTSC-OMe** complex and (ii) the great difference in the molecular absorptivity (ε) of complexes. Concerning the first hypothesis, since the reductive quenching process of photosensitizer by the catalyst is in competition with the expected reaction with TEA, decreasing the catalyst concentration will possibly favor the suggested reductive pathway. Moreover, regarding the second probable assumption, in our previous work the photosensitizer [**ZnTMePy]**^**+**^ exhibited an absorption coefficient of ε = 1,80,000 M^−1^.cm^−1^. In the present study though, the **Ps1** exhibits an absorption coefficient of ε = 6000 M^−1^.cm^−1^ (Andreiadis et al., 2011), and the catalyst (**NiTSC-OMe**) displays an absorption band at 470 nm with an epsilon value of ε = 17,000 M^−1^cm^−1^ (Straistari et al., 2017). Consequently, the absorption properties of the catalyst can reduce the available photons for the photosensitizer, thus the catalyst concentration should be lower than that of the photosensitizer in order for the system to be more efficient.

**Figure 4:**
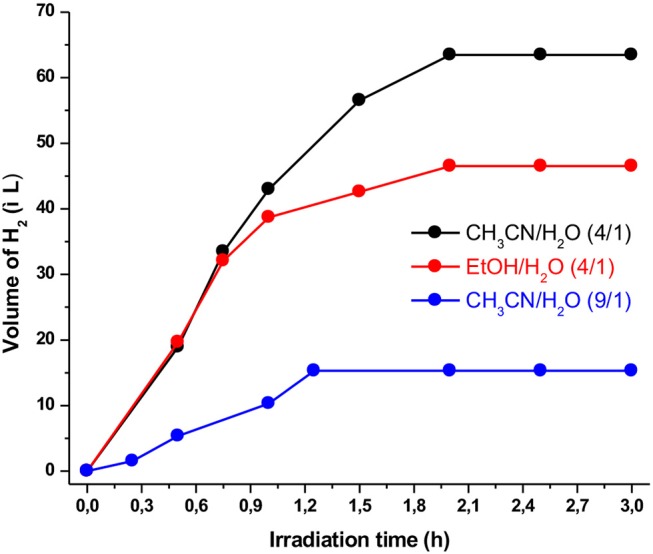
Effect of various solvent mixtures on hydrogen production using the photocatalytic system containing **Ps1** (5 × 10^−4^ M), **NiTSC-OMe** (5 × 10^−8^ M), and TEA [5% (v/v)] at pH 10.

The nature as well as the ratio of the solvents definitely plays a significant role in the HER. As a result, we examined two different solvent mixtures, i.e., CH_3_CN/H_2_O and Ethanol/H_2_O, using also different ratio (from 4:1 to 9:1). As shown in Figure 4, the best solvent mixture was found to be CH_3_CN/H_2_O in a 4:1 ratio producing a maximum volume of hydrogen of 63 μL after almost 2 h of irradiation. We concluded that the solvent ratio induced critical changes on the hydrogen production, most likely because affecting the solubility properties. These solubility properties can be altered via the dielectric constant and the diffusion coefficient of each solvent (Rao et al., 2015; Pellegrin and Odobel, 2017). When CH_3_CN/H_2_O in a 9:1 ratio was utilized as the solvent mixture, the photocatalytic performance dramatically decreased. The smaller water concentration probably leads to lower solubility with direct impact on the photocatalytic activity of the system.

**Figure 5:**
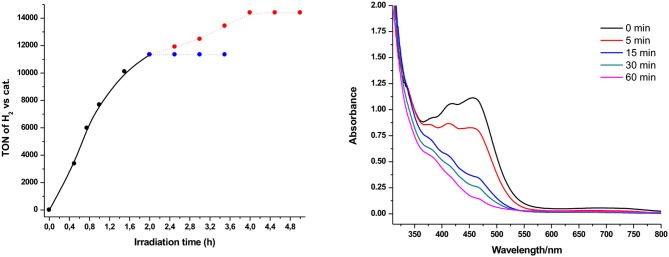
Hydrogen production upon irradiation of solutions (4:1 CH_3_CN/H_2_O) containing **Ps1** (5 × 10^−4^ M), **NiTSC-OMe** (5 × 10^−8^ M), and TEA [5% (v/v)] at pH = 10. Regeneration of **Ps1** and **NiTSC-OMe** (red dot line). Regeneration of **Ps1** or **NiTSC-OMe** (blue dot line) **(left)**. UV-vis absorption spectra recorded during the course of the photolysis experiment in 10 mm cuvette, containing **Ps1** (7 × 10^−5^ M), **NiTSC-OMe** (5 × 10^−5^ M), and TEA [5% (v/v)] at pH = 10 **(right)**.

In all presented photocatalytic experiments, H_2_ production stops after almost 2 h of irradiation. Therefore, regeneration and photolysis experiments have been performed in order to examine the stability of our system (Figure 5 and Figure S3). As illustrated in Figure 5 (right part), we performed UV-Vis absorption photolysis experiment in a solution containing **Ps1** and **NiTSC-OMe**. The characteristic absorption bands of our system (416 for **Ps1** and 460 nm for **NiTSC-OMe**) were significantly decreased after 15 min of irradiation, suggesting bleaching of the photolysis solutions. After the addition of either the catalyst or the photosensitizer we didn't observed any hydrogen evolution. However, when both components were added to the reaction mixture, the catalytic system was effectively regenerated, leading to 3,067 TON (Figure 5, left), suggesting that both these components undergo concomitant decomposition after almost 2 h of irradiation.

The degraded compounds can form metallic nanoparticles, which can act as the catalytic species during HER (Lazarides et al., 2014). In order to exclude this possibility we performed mercury poisoning experiments. Photocatalytic experiments in the presence of mercury showed no significant change in the amount of the produced H_2_ (11,787 TONs), thus confirming the homogeneous nature of our photocatalytic system.

To shed light into the mechanism of photocatalytic system, fluorescence spectroscopy was used. Specifically, emission spectroscopy experiments were carried out using **Ps1** (4 × 10^−5^ M) as photosensitizer upon its excitation at 337 nm in CH_3_CN solution. While the photocatalytic measurements were performed in a CH_3_CN/H_2_O mixture, the photophysical studies were carried out using CH_3_CN as a solvent. This is due to the fact that TEA was not soluble enough in the high concentrations needed for the Stern Volmer plots in the CH_3_CN/H_2_O mixture. Firstly, we examined the quenching process on **Ps1** by increasing the concentration of either TEA (0 → 0.36 M)] (Figure S5, left) or **NiTSC-OMe** (0 → 4.1 × 10^−5^ M) (Figure S6, left). In addition, we have calculated the Stern-Volmer constant (K_SV_) based on Stern-Volmer plot (Figures S4, S5, right), using the equation I_o_/I = 1 + K_SV_[Q], where I_o_ and I are the fluorescence intensities observed in the absence and in the presence of each quencher, respectively, and [Q] is defined as the quencher concentration (Keizer, 1983). In perfect agreement with previous publications, the K_SV_ constant was higher in the case of the catalyst (K_SV_ = 13,419.5 M^−1^) compared to the TEA (K_SV_ = 11.7 M^−1^). Moreover, we calculated the quenching constant for **NiTSC-OMe** (K_Q_ = 4.99 × 10^10^ M^−1^s^−1^) and for TEA (K_Q_ = 4.35 × 10^7^ M^−1^s^−1^) as well, which are derived from the equation: K_SV_ = K_Q_ τ, where τ stands for the excited state lifetime in the absence of the quencher (Han et al., 2013; Yuan et al., 2016). However, under similar conditions to the H_2_ production experiments in the emission spectra of **Ps1**, we observe that the characteristic fluorescence peak of the photosensitizer (at 590 nm) decreases only when the sacrificial electron donor (TEA) is added (Figure S7). What is more, there is a great difference in the quenching process, namely 50% in case of TEA and 1% in case of the catalyst. Therefore, even though, the rate constant by the **NiTSC-OMe** is greater than the rate constant by TEA, the major electron transfer pathway occurs from the excited photosensitizer to TEA, since the concentration of electron donor is bigger than that of the catalyst in the system (Han et al., 2013). In summary, hydrogen production in our system is initiated via reductive quenching of the photosensitizer.

The redox potentials of the reported compounds used in this study are listed in Table 1. Based on these values the thermodynamic driving forces for the different electron transfer processes, ΔG^i^(PS/Cat), were calculated [(Queyriaux et al., 2017) and references herein, Goldsmith et al., 2005]. The resulting ΔG^1^(PS/Cat) values turn out to be in agreement with the fluorescence quenching measurements which seems to indicate that the initial step is the reductive quenching of the photosensitizer. Indeed, the calculated ΔG^1^(PS/Cat) values are positive for a reduction from Ps^+^/Ps^*^ for **Ps1** and **Ps2** (+0.44 and +0.18, respectively) whereas they are negative for a reduction from Ps/Ps^−^ (−0.13 and −0.24, respectively). It is also possible to find that whatever the mechanism is, the **Ps3** potentials are not negative enough to reduce the nickel catalyst, which would explain the observed lack of photocatalytic activity. The double reduction of the catalyst is also unlikely since the values of ΔG^2^(PS/Cat) are largely positive (> +0.40 eV) which would not be in favor an EECC mechanism (E corresponds to an electron transfer step and C to a chemical reaction, here protonation). Finally, the difference in activity between **Ps1** and **Ps2** does not seem to be rationalized by the little difference in driving force of injection (respectively, −0.13 and −0.24 eV), but rather by the difference of driving regeneration force ΔG(SED/PS) which is larger in the case of **Ps2** than for **Ps1** (respectively, +0.09 and 0 eV).

**Table 1:** Redox potentials (V vs. NHE) of the different compounds employed in this study (Ps* represents the excited state of Ps: Ps^+^ and Ps^−^, its oxidized and reduced forms, respectively) together with the thermodynamic driving forces for the different electron transfer processes (ΔG^1^(PS/Cat), ΔG^2^(PS/Cat), and ΔG(SED/PS), eV).

**Photosensitizers**	E(Ps^**+**^/Ps*)	**E(Ps/Ps^**−**^)**	**E(Ps^**+**^/Ps)**	E(Ps*/Ps^**−**^)
**Ps1**	−0.60	−1.17	+1.50	+0.93
ΔG^1^(PS/Cat)	+0.44	−0.13		
ΔG^2^(PS/Cat)	+1.07	+0.50		
ΔG(SED/PS)			−0.57	0
**Ps2**	−0.86	−1.28	+1.26	+0.84
ΔG^1^(PS/Cat)	+0.18	−0.24		
ΔG^2^(PS/Cat)	+0.81	+0.39		
ΔG(SED/PS)			−0.33	+0.09
**Ps3**	−0.45	−0.85	+1.18	+0.78
ΔG^1^(PS/Cat)	+0.26	+0.20		
ΔG^2^(PS/Cat)	+1.22	+0.82		
ΔG(SED/PS)			−0.25	+0.15
**Catalyst**	**E(Ni**^**II**^**/Ni**^**I**^**)**	**E(Ni**^**I**^**/L**^**·−**^**)**	**SED**	**E**_**O**__x_
**NiTSC-OMe**	−1.04	−1.67	**TEA**	0.93

**Scheme 1:**
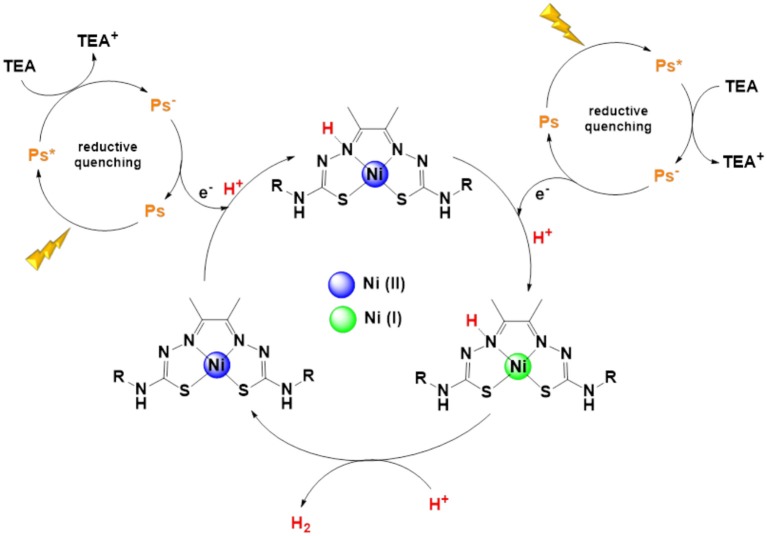
Proposed mechanism of hydrogen photogeneration.

Taking into consideration all studies reported in this work, we propose a possible H_2_ production mechanism as illustrated in Scheme 1. First, [Ir(ppy)_2_(bpy)]^+^ (Ps) is excited by visible light irradiation to form the excited state of the photosensitizer (Ps^*^). Subsequently, the sacrificial electron donor (TEA) transfers an electron to the excited photosensitizer via reductive quenching process forming its oxidized state (TEA^+^) and the reduced state of the photosensitizer (Ps^−^) (Scheme 1).

Then, a protonation of coordinated *N*-atom of the ligand on nickel complex takes place (Straistari et al., 2017) followed by an electron transfer process from the photosensitizer (Ps^−^) to the nickel catalyst, creating the nickel (I) complex and forming an hydride intermediate as presented in the scheme bellow. Finally, H_2_ production from the system through the nickel catalyst occurs together with its regeneration.

## Conclusions

In summary, a systematic photocatalytic study toward light-driven hydrogen production is presented herein, using three different chromophores and one bis-thiosemicarbazone nickel complex. When **NiTSC-OMe** was combined with either **Ps2** or **Ps3**, no hydrogen production was observed under various experimental conditions. However, when Ps1 was utilized as a chromophore, H_2_ production was detected. In order to estimate the optimal conditions we examined the influence of various parameters: the concentration of the catalyst, the pH value of the buffer solution and the ratio as well as the nature of the solvent mixture. Overall, the highest amount of H_2_ (204 μL,) was attained using a 4:1 CH_3_CN/H_2_O solution containing **NiTSC-OMe** (10^−6^ M), **Ps1** (5 × 10^−4^ M), TEA [5% (v/v)] at pH 10. The maximum catalytic activity of the system though, with the highest TON and TOF values (11,333 and 7,971) were observed using **NiTSC-OMe** (5 × 10^−8^ M), **Ps1** (5 × 10^−4^ M), TEA [5% (v/v)] at pH 10 in 4:1 CH_3_CN/H_2_O solution. All the promising results displayed in this study offer new aspects regarding the combination of chromophores with nickel catalysts for hydrogen production. In addition, such efforts could in general provide new perspectives improving the efficiency and the function of catalytic systems developed for photoinduced hydrogen evolution using water.

## Author Contributions

MO and AC designed and directed the study. AP synthesized the catalyst. SP, GL, and VN performed the experiments. RH, JM, GC, AC, and MO analyzed the data. GC, AC, and MO wrote the paper with input from all authors. All authors contributed to the design and implementation of the research, to the analysis of the results and to the writing of the manuscript.

### Conflict of Interest Statement

The authors declare that the research was conducted in the absence of any commercial or financial relationships that could be construed as a potential conflict of interest.
