# Supplementary material for: Efficient Light-Driven Hydrogen Evolution Using a Thiosemicarbazone-Nickel (II) Complex
Source: Front Chem. 2019 Jun 27;7:405. doi: 10.3389/fchem.2019.00405 (PMC6610430; doi:10.3389/fchem.2019.00405)
Supplement: Supplementary file 1 [file Data_Sheet_1.pdf]

Supplementary Material

Efficient light-driven Hydrogen evolution using a Thiosemicarbazone-Nickel (II) complex.

Stylianos Panagiotakis,[1] Georgios Landrou,[1] Vasilis Nikolaou,[1] Anisa Putri,[2] Renaud Hardré,[2] Julien Massin,[2] Georgios Charalambidis,[1]* Athanassios G. Coutsolelos,[1]* Maylis Orio[2]*

[1] Laboratory of Bioinorganic Chemistry, Department of Chemistry, University of Crete, Voutes Campus, 70013 Heraklion, Crete, Greece.

[2] Aix Marseille Univ, CNRS, Centrale Marseille, iSm2, Marseille, France

*** Correspondence:**
Corresponding Authors
email: gcharal@uoc.gr
email: acoutsol@uoc.gr
email: maylis.orio@univ-amu.fr

1- Supplementary Figures

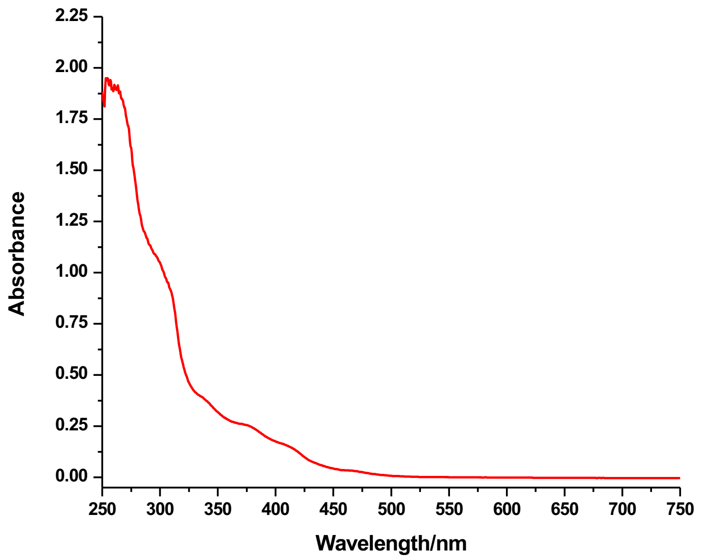

Figure S1: UV-Vis absorption spectra containing **Ps1**, TEA [5% (v/v)] in a 4:1 $CH_3CN:H_2O$ solution at pH=10.

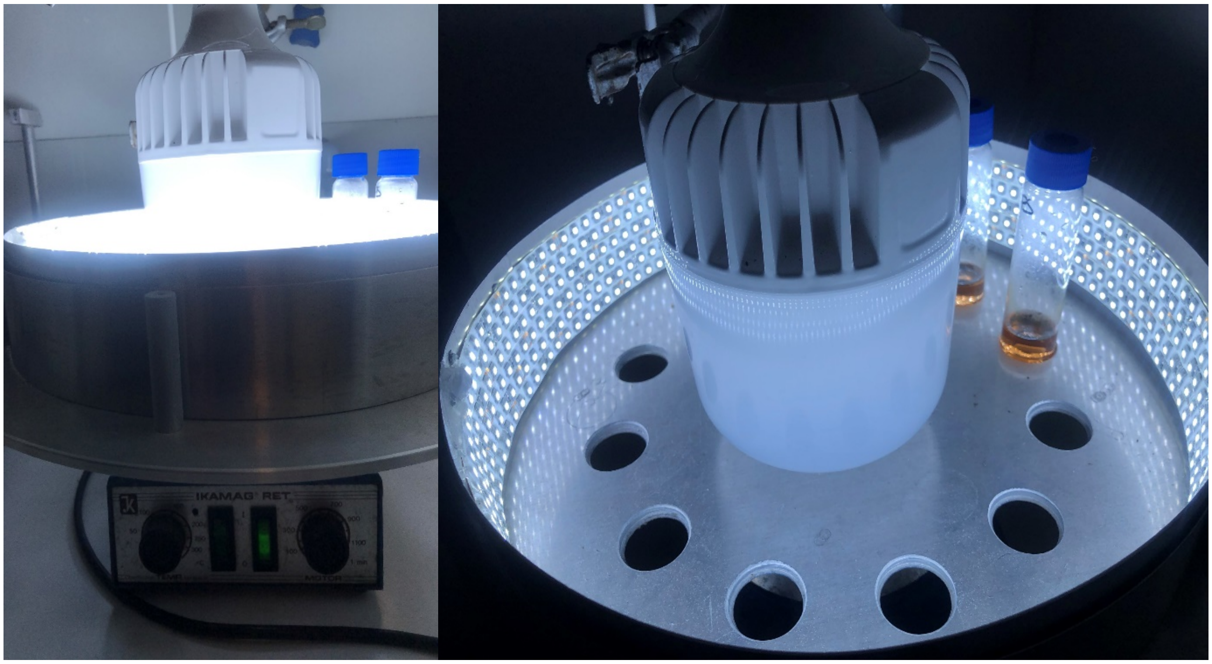

Figure S2: The experimental set-up using the LED lamp.

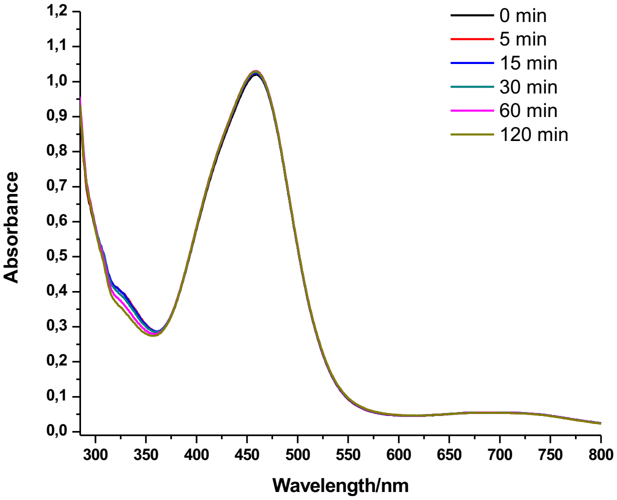

Figure S3: UV-Vis absorption spectra recorded during the course of the photolysis experiment containing $5x10^{-5}$ M **NiTSC-OMe**, TEA [5% (v/v)] in a 4:1 CH_3CN:H_2O solution at pH=10.

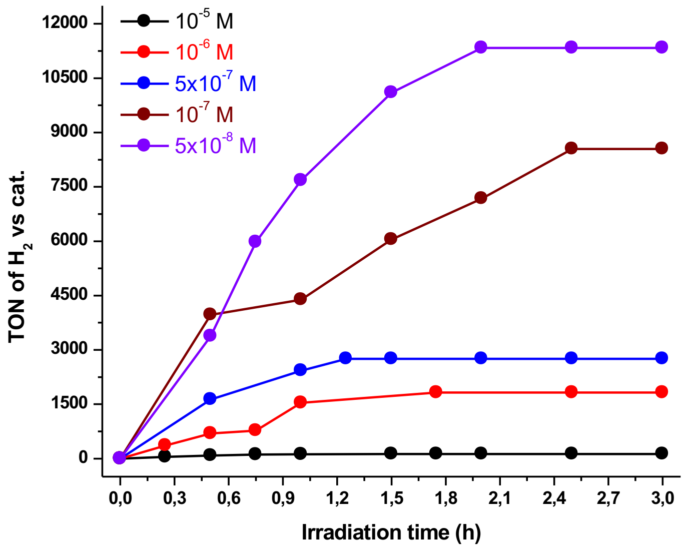

Figure S4: Plot of Turnover Numbers (TONs) upon irradiation of different **NiTSC-OMe** concentrations containing 5x10^{-4} M of **Ps1**, TEA [5% (v/v)] in a 4:1 CH_3CN/H_2O solution at pH 10.

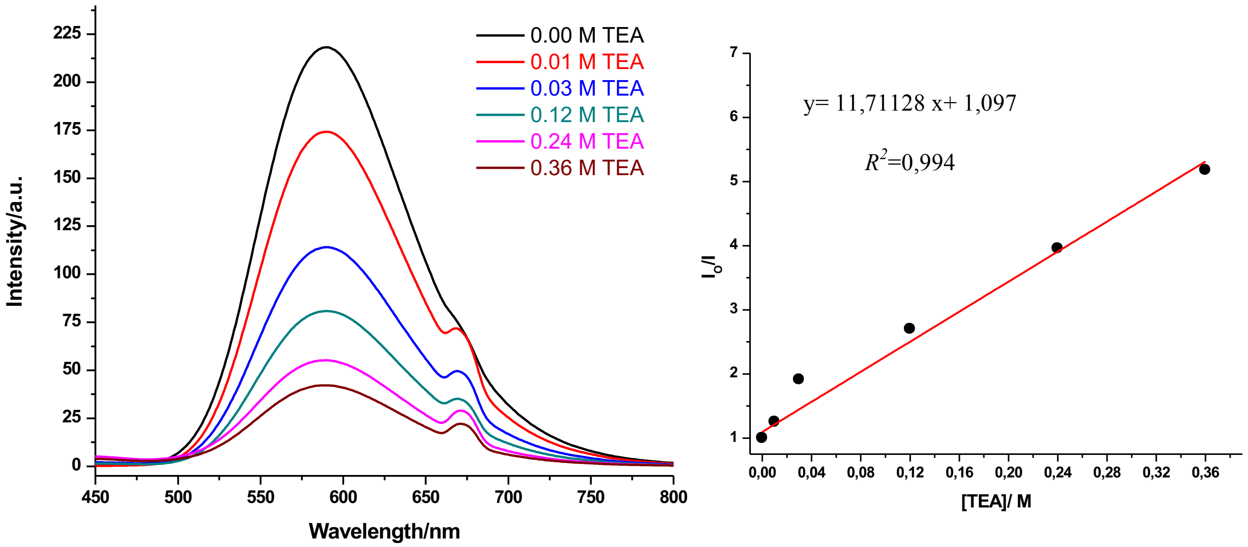

Figure S5: Emission quenching spectra of **Ps1** solution (4x10^{-5} M) by **TEA** when excited at 337 nm in CH_3CN solution (left). Stern-Volmer plot of emission quenching (right).

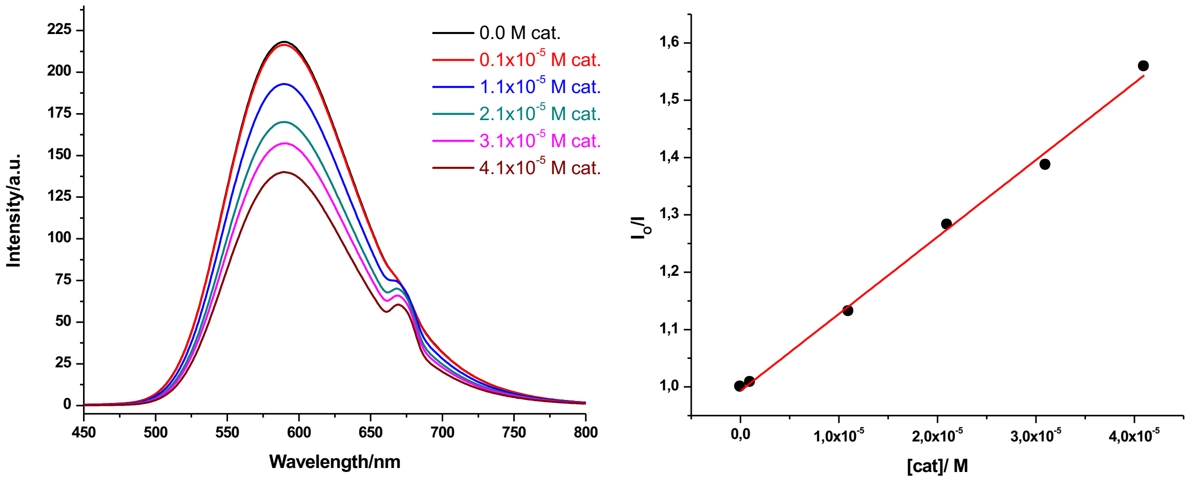

Figure S6: Emission quenching spectra of **Ps1** solution ($4x10^{-5}$ M) by **NiTSC-OMe** when excited at 337 nm in CH_3CN solution (left). Stern-Volmer plot of emission quenching (right).

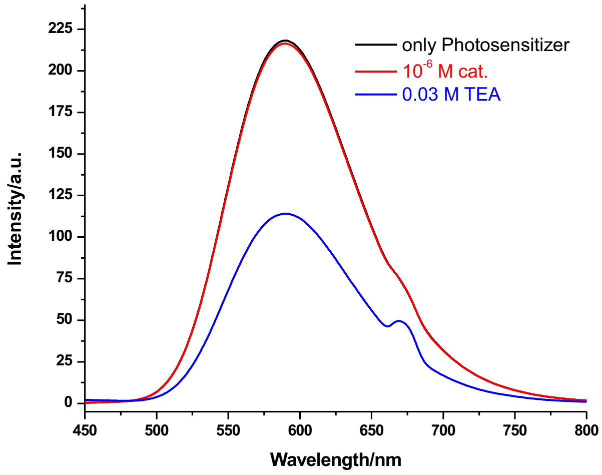

Figure S7: Emission quenching spectra of **Ps1** solution ($4x10^{-5}$ M) (black line) by **NiTSC-OMe** (red line) and TEA (blue line) when excited at 337 nm under similar photocatalytic conditions.

2- Supplementary Tables

Table S1. Variation of the photocatalytic activity using different concentrations of **NiTSC-OMe**. Conditions: samples under investigation contain $5x10^{-4}$ M of **Ps1**, TEA [5% (v/v)] in a 4:1 CH_3CN/H_2O solution at pH 10.

Cat	C_{Cat} (M)	Photosensitizer	C_{PS} (M)	H_2 (µmol)	H_2 (µL)	TON	TOF (h^{-1}) (max)
NiTSC-OMe	10^{-5}	$[Ir(ppy)_2(bpy)]^+$	$5*10^{-4}$	6.3	140	125	83 (183)
NiTSC-OMe	10^{-6}	$[Ir(ppy)_2(bpy)]^+$	$5*10^{-4}$	9.1	204	1821	1040 (1538)
NiTSC-OMe	$5x10^{-7}$	$[Ir(ppy)_2(bpy)]^+$	$5*10^{-4}$	6.9	154	2748	2199 (2423)
NiTSC-OMe	10^{-7}	$[Ir(ppy)_2(bpy)]^+$	$5*10^{-4}$	4.3	96	8542	3417 (7938)
NiTSC-OMe	$5x10^{-8}$	$[Ir(ppy)_2(bpy)]^+$	$5*10^{-4}$	2.8	63	11333	5667 (7971)
